# Accuracy of duplex ultrasound in peripheral artery disease: a systematic review and meta-analysis

**DOI:** 10.1590/1677-5449.202400332

**Published:** 2025-02-14

**Authors:** Silfayner Victor Mathias Dias, Ronald Luiz Gomes Flumignan, Nelson Carvas, Wagner Iared

**Affiliations:** 1 Universidade Federal de São Paulo – UNIFESP, Escola Paulista de Medicina – EPM, São Paulo, SP, Brasil.

**Keywords:** arterial occlusive diseases, diagnostic imaging, lower extremity, peripheral vascular diseases, ultrasonography, doença arterial oclusiva, imagem diagnóstica, membro inferior, doença vascular periférica, ultrassonografia

## Abstract

Lower limb peripheral artery disease (PAD) is highly prevalent. Current guidelines recommend duplex ultrasound (DUS) with spectral analysis for diagnosis. This systematic review and meta-analysis assessed the diagnostic accuracy of DUS in symptomatic PAD patients. We searched electronic databases for studies comparing DUS and arteriography. Arteries were analyzed individually and grouped into segments (aorto-common femoral, femoropopliteal, below the knee, and the entire lower limb). The meta-analysis estimated sensitivity, specificity, likelihood, and diagnostic odds ratios (DOR). Fifteen studies were included, analyzing 9,067 arteries. DUS accuracy for symptomatic PAD was 0.86 (95% CI 0.81-0.90) for sensitivity and 0.95 (95% CI 0.78-0.97) for specificity. The best results were observed for the femoropopliteal segment: sensitivity 0.86 (95% CI 0.80-0.90), specificity 0.95 (95% CI 0.93-0.97). The poorest performance was observed for the below-the-knee segment: sensitivity 0.78 (95% CI 0.60-0.89), specificity 0.92 (95% CI 0.78-0.97). Most studies had high and unclear risk of bias. There is significant heterogeneity in results, with a limited number of primary studies for each arterial segment, especially for the below-the-knee segment.

## Resumo

A doença arterial periférica (DAP) nos membros inferiores é altamente prevalente, e as diretrizes atuais recomendam o uso do ultrassom Doppler (UD) com análise espectral para diagnóstico. Esta revisão sistemática e metanálise avaliou a acurácia diagnóstica do UD em pacientes sintomáticos com DAP. Foram pesquisados estudos em bancos de dados eletrônicos que comparassem UD e arteriografia. As artérias foram analisadas individualmente e agrupadas em segmentos: aortofemoral comum, femoropoplíteo, abaixo do joelho e todo o membro inferior. A metanálise estimou sensibilidade, especificidade, verossimilhança e razão de chances diagnóstica (DOR). Quinze estudos foram incluídos, analisando 9.067 artérias. A acurácia do UD para DAP sintomática foi de 0,86 (intervalo de confiança de 95% [IC 95%] 0,81-0,90) para sensibilidade e 0,95 (IC 95% 0,78-0,97) para especificidade. O segmento femoropoplíteo apresentou os melhores resultados, com sensibilidade de 0,86 (IC 95% 0,80-0,90) e especificidade de 0,95 (IC 95% 0,93-0,97). O segmento abaixo do joelho teve o pior desempenho, com sensibilidade de 0,78 (IC 95% 0,60-0,89) e especificidade de 0,92 (IC 95% 0,78-0,97). A maioria dos estudos apresentou risco de viés alto e incerto. Observou-se heterogeneidade significativa nos resultados, com número limitado de estudos primário para cada artéria, especialmente para o segmento abaixo do joelho.

## INTRODUCTION

Peripheral arterial disease (PAD) is a chronic obstructive disease characterized by progressive narrowing of the arterial lumen (stenosis) attributed to atherosclerotic plaques. In advanced cases, occlusion of the vessel may occur.^1^ The reported prevalence of lower limb PAD is 18% in patients over 50 years of age, reaching 29% in those over 70. Incidence has been increasing over the decades related to the growth and aging of the population, diabetes mellitus, and smoking.^2^ Current guidelines recommend a duplex ultrasound (DUS) examination as an initial complementary test to confirm and localize the disease.^3^

DUS has many advantages. It is widely available, can be done at the bedside, is non-invasive, and does not require ionizing radiation or contrast media. However, it has limitations. The results depend on the operator’s skill, visualizing deep anatomical structures can be challenging, and issues may arise due to intestinal gas or arterial wall calcifications.^4^

Arteriography is the current standard for diagnosing PAD, but it is invasive and uses contrast media and ionizing radiation. There are two other diagnostic tests available: computed tomography (CT) angiography and magnetic resonance (MR) angiography. CT angiography is generally preferred, while MR angiography can sometimes struggle to distinguish between arteries and veins. Both methods use contrast-based diagnostic images. All three tests evaluate stenoses and occlusions by showing a reduction in the vessel lumen, which differs from DUS, which assesses blood flow speed.

Only one systematic review, conducted by Collins et al.,^5^ has evaluated the accuracy of DUS for diagnosing PAD. However, the studies included in that review considered only B Mode and Color Doppler findings as criteria for diagnosing stenosis and occlusion. Current guidelines recommend the incorporation of spectral Doppler for diagnosis.^6^

Using three DUS imaging modalities (B Mode, Color Doppler, and Spectral Doppler) improves the reliability of diagnosis of arterial stenosis and occlusions. Color Doppler displays blood velocity using color, showing the average of the highest velocities in each screen pixel for a specific vessel segment. This color-coded representation helps identify the reference point for performing spectral analysis. At this point, Spectral Doppler provides a graphical representation of blood flow velocity over time.^7^

This systematic review and meta-analysis aimed to determine the diagnostic accuracy of DUS, including spectral analysis, for symptomatic patients with lower limb PAD.

## METHODS

This systematic review was registered on the International Prospective Register of Systematic Reviews (CRD42017056299). It was conducted following the criteria established in the PRISMA (Preferred Reporting Items for Systematic Reviews and Meta-Analyses) statement.^8^

### Search strategy and study eligibility criteria

The searches were conducted on MEDLINE via PubMed, Embase, the Latin American and Caribbean Health Sciences Literature database, the Spanish-Language Bibliographic Index for Health Sciences, and the Web of Science databases, as well as on the Cochrane Central Register of Controlled Trials, ClinicalTrials.gov, and the World Health Organization trial registries. We imposed no restrictions regarding the date of publication or language. We included only studies evaluating the diagnostic accuracy of DUS for arterial stenosis and occlusion, compared with arteriography (the reference test), in symptomatic patients with PAD.

Case-control studies were excluded because they often overestimate a test’s accuracy in clinical practice. This bias occurs because these studies typically include patients who already have an established diagnosis, which can artificially inflate sensitivity and specificity compared to other study designs. By excluding case-control studies, we eliminated a significant source of bias to better reflect the diagnostic test’s performance.^9^

Hemodynamically significant stenosis (≥ 50% reduction in vessel diameter) was evaluated, characterized by at least twice the flow velocity at the point of most significant narrowing in relation to the regular proximal segments. Occlusion was defined as the absence of flow on spectral analysis. We employed the Quality Assessment of Diagnostic Accuracy Studies, version 2 (QUADAS-2),^10,11^ to assess the risk of bias and the applicability of the included studies.

### Statistical analysis and data synthesis

This systematic review used primary studies to conduct two separate topographic analyses. The first analysis focused on specific segments: aorto-common femoral, femoropopliteal, and below the knee. The segment below the knee includes the tibiofibular trunk and the anterior, posterior tibial, and fibular arteries. Finally, we evaluated the entire lower limb collectively.

Statistical analyses were performed following the recommendations outlined in the Cochrane Handbook for Systematic Reviews of Diagnostic Test Accuracy.^12^ We inspected forest plots to determine the direction, magnitude of effects, degree of overlap between confidence intervals, and heterogeneity.^13^ The random-effects model was used, considering the potential heterogeneity of the studies. When there was substantial heterogeneity, the probable causes were explored by analyses of subgroups.

High heterogeneity in systematic reviews of diagnostic test accuracy can result from several factors, including disease severity and prevalence, personnel expertise variations, study design differences, reference standard variability, publication bias, statistical analysis methods, and how results are reported and interpreted.

Diagnostic accuracy statistics were computed using R software version 4.0.0 (R Core Team). The results for sensitivity, specificity, positive and negative likelihood ratio, and DOR (diagnostic odds ratio) are presented with 95% confidence intervals.

### Sensitivity analysis

We planned a sensitivity analysis for each artery included in this review, investigating the effect of excluding studies with an uncertain or high risk of bias. In those analyses, we considered each of the domains that make up the QUADAS-2 risk of bias assessment.

There is currently no uniformly accepted and validated method for analysis of publication bias for systematic reviews of diagnostic accuracy testing.^14^

Patients or the public were not involved in the design, conduct, reporting, or dissemination plans of this trial.

## RESULTS

### Literature search

**Figure 1:**
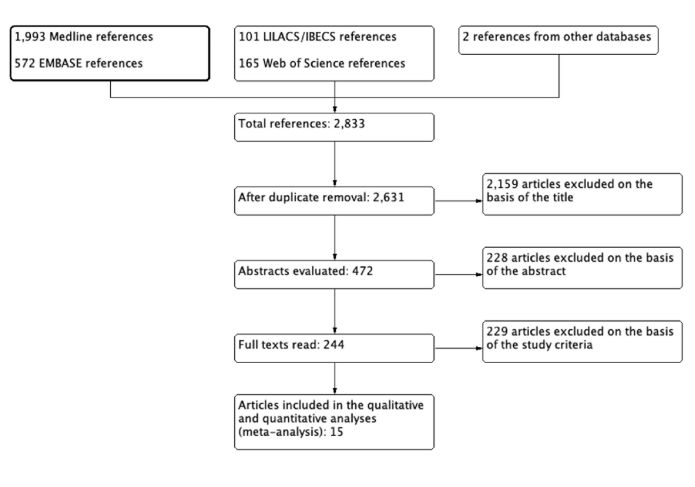
PRISMA flow diagram of the study selection process. LILACS, *Literatura Latinoamericana y del Caribe en Ciencias de la Salud* (Latin-American and Caribbean Health Sciences Literature); IBECS, *Índice Bibliográfico Español en Ciencias de la Salud* (Spanish-Language Bibliographic Index for Health Sciences); PRISMA, Preferred Reporting Items for Systematic Reviews and Meta-Analyses.

All electronic searches were performed in May 2020 and updated in March 2022, and 2,833 records were retrieved (Figure 1). After removal of duplicates, 2,631 records remained. After reviewing the titles and abstracts, 2,387 studies were deemed irrelevant, and the full texts of the remaining 244 studies were evaluated. Of those 244 studies, 229 were excluded. Therefore, 15 studies met the inclusion criteria for qualitative and quantitative assessment. The characteristics of the included studies are detailed in Table 1.

**Table 1:** Characteristics of the included studies.

**Reference**	**Year**	**Study design**	**Disease Severity [13]**	**Arteries evaluated**
Bergamini et al.^15^	1995	Prospective	Claudication and severe ischemia	CF, P3F, D3F, SAPOP, IAPOP, TTF
Cossman et al.^16^	1989	Retrospective	Claudication and severe ischemia	I, CF, P3F, D3F, SAPOP, IAPOP, TTF
Fletcher et al.^17^	1990	Prospective	Claudication and severe ischemia	P3F, M3F, D3F
Gjønnæss et al.^18^	2006	Prospective	Claudication	FS
Jager et al.^19^	1985	Prospective	Claudication and severe ischemia	I, CF, P3F, M3F, D3F, POP
Karacagil et al.^20^	1996	Prospective	Claudication and severe ischemia	IAPOP, AT, PT, FIB
Kohler et al.^21^	1987	Prospective	Not reported	A, I, CF, M3F, POP
Lai et al.^22^	1996	Prospective	Not reported	A, CI, EI, CF, P3F, M3F, D3F, SAPOP
Larch et al.^23^	1997	Prospective	Claudication and severe ischemia	AT, PT, FIB
Moneta et al.^24^	1992	Prospective	Claudication and severe ischemia	I, CF, FS, POP, AT, PT, FIB
Moreira^25^	2009	Prospective	Claudication and severe ischemia	A, CI, EI
Pinto et al.^26^	1996	Prospective	Claudication and severe ischemia	P3F, D3F, SAPOP, IAPOP
Polak et al.^27^	1990	Prospective	Claudication and severe ischemia	P3F, M3F, D3F, IAPOP, SAPOP
Whelan et al.^28^	1992	Prospective	Claudication and severe ischemia	CF, P3F, D3F, SAPOP, IAPOP, TTF
Zeuchner et al.^29^	1994	Prospective	Claudication and severe ischemia	CF, I

Legend: A, aorta; I, iliac; CI, common iliac; EI, external iliac; CF, common femoral; F, femoral; P3F, proximal third of the femoral; M3F, middle third of the femoral; D3F, distal third of the femoral; POP, popliteal; SAPOP, supra-articular popliteal; IAPOP, infra-articular popliteal; TFT, tibiofibular trunk; AT, anterior tibial; PT, posterior tibial; FIB, fibular.

### Assessment of the risk of bias

**Figure 2:**
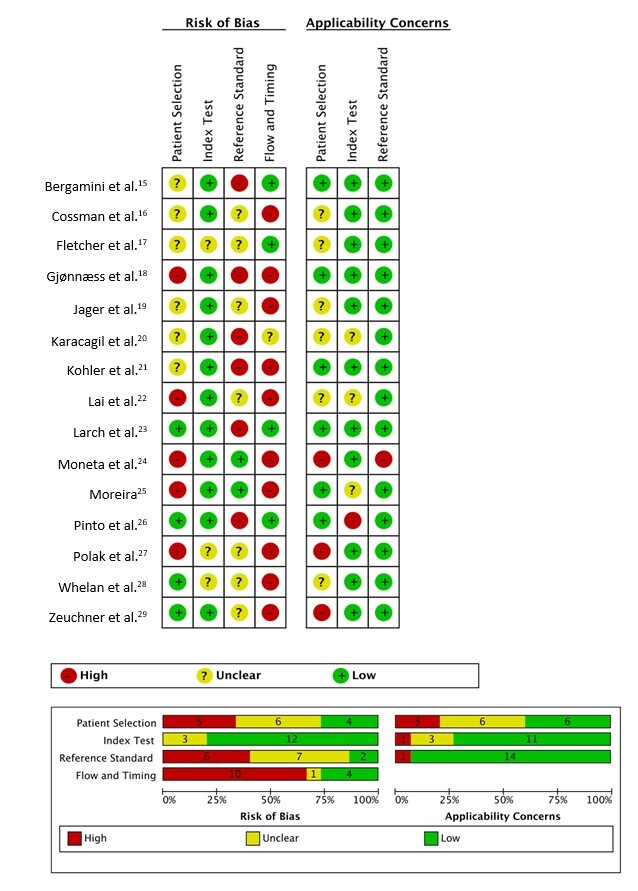
Risk of bias and applicability concerns for each domain of the Quality Assessment of Diagnostic Accuracy Studies, version 2: summary and graphic.

All articles included in this review were evaluated with QUADAS-2 and the results are summarized in Figure 2. Five of the 15 studies evaluated (33%) did not employ appropriate patient selection and six (40%) failed to provide sufficient details of the patient selection process. Two studies (27%) did not classify patients by disease severity. All the authors evaluated the index test independently of the reference test results. In thirteen studies (87%), appropriately qualified professionals performed the index test. The two other studies did not report sufficient data regarding performed of the index test. All the studies used arteriography as the reference test. Single-plane arteriography was used in six studies (40%), biplanar was used in two (13%), and seven (47%) failed to provide details about the arteriography. All studies evaluated the reference test results independently of those of the index test. The interval between the index test and the reference test was less than 30 days in twelve studies (80%) and ≤ 12 months in three (20%). Arterial segments were not analyzed as proposed in eleven studies (73%). Among those, the median proportion of arteries not analyzed was 5%. The reasons for not analyzing segments were a minimal quantity of contrast medium administered (limited by the clinical condition of the patients), amputations, and failure to visualize specific arteries with DUS.

Data from the risk of bias assessment help interpret variability and confounding factors in the systematic review results. We noted that many primary studies had a high or uncertain risk of bias in patient selection, using reference standards, and analyzing the initially proposed arteries. These assessment results explain the wide confidence intervals in the data analyses. These risks are crucial when applying these findings to each evaluated segment.

The sixteen arteries proposed were included in the analysis by segments – aorto-common femoral, femoropopliteal, and below the knee – and for the entire lower limb analysis. Table 2 summarizes the results.

**Table 2:** Analysis.

**Segment**	**Ar/St**	**Sens**	**95% CI**	**Spec**	**95% CI**	**LR+**	**95% CI**	**LR−**	**95% CI**	**DOR**	**95% IC**
**Entire lower limb**	9067/15	0.86	(0.81–0.90)	0.95	(0.90–0.97)	17.20	(9.88-30.53)	0.15	(0.11-0.20)	4.79	(4.11-5.47)
**Aorto-common femoral**	1924/9	0.78	(0.68-0.85)	0.96	(0.93-0.98)	19.50	(13.02-43.67)	0.23	(0.13-0.34)	4.67	(3.81-5.53)
**Femoropopliteal**	5000/12	0,86	(0.80–0.90)	0.95	(0.93–0.97)	17.20	(12.43-35.12)	0.15	(0.10-0.19)	5.10	(4.28-5.92)
**Below the knee**	2143/5	0.78	(0.60–0.89)	0.92	(0.78–0.97)	6,82	(1.97-18.57)	0,28	(0.12-0.65)	3.24	(1.84-4.63)

Legend: Ar, (N of) arteries; St, (N of) studies; Sens, sensitivity; CI, confidence interval; Spec, specificity; LR+, positive likelihood ratio; LR−, negative likelihood ratio; DOR, diagnostic odds ratio.

**Figure 3:**
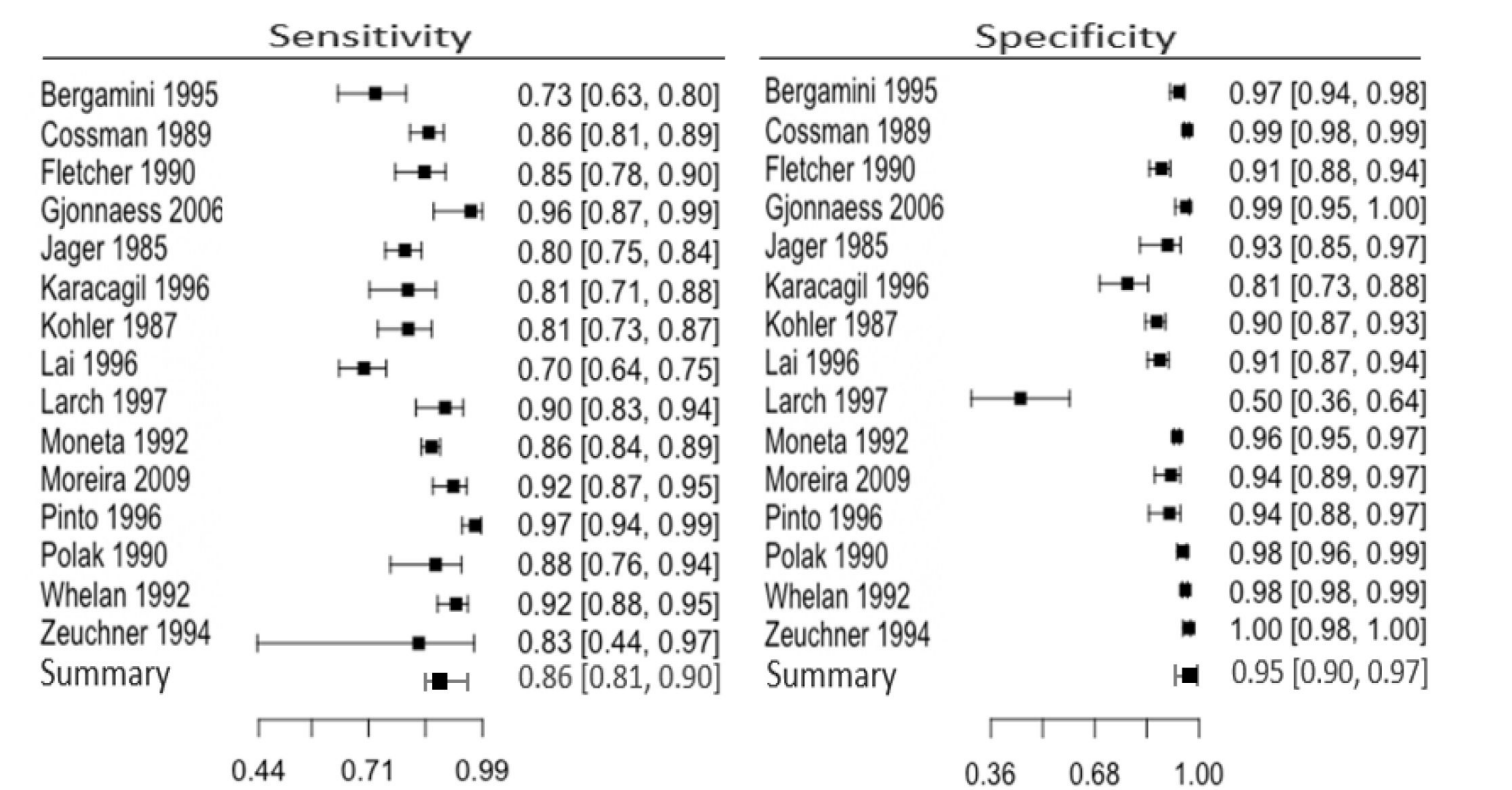
Results of the meta-analysis for the entire lower limb.

Based on the meta-analysis of 9067 arteries in symptomatic patients with PAD, we concluded that the sensitivity and specificity of DUS for the diagnosis of arterial lesions in the entire lower limb were respectively 0.86 (95% CI: 0.81-0.90) and 0.95 (95% CI: 0.90-0.97). Visual analysis of the forest plot demonstrates more significant heterogeneity and wider confidence intervals for sensitivity than specificity (Figure 3).

**Figure 4:**
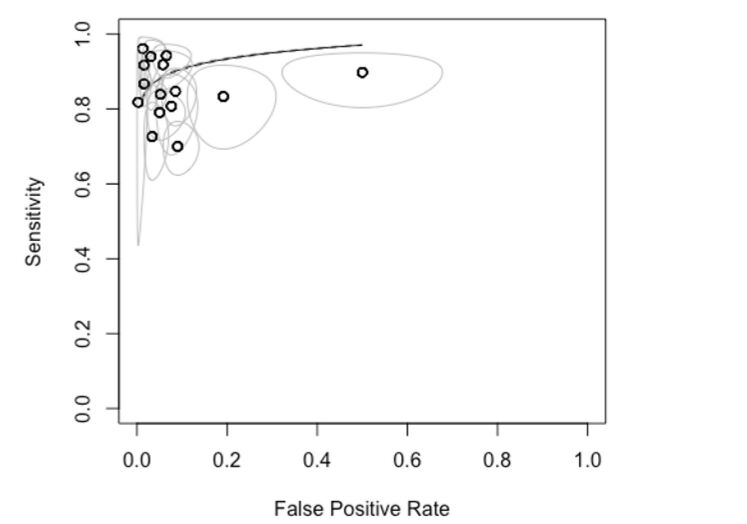
SROC for analysis of all comparisons in the study.

In the SROC (summary receiver operating characteristic) graph, we observed that most primary studies are positioned in the upper left quadrant, indicating high sensitivity and specificity values (Figure 4). Each study in this quadrant tends to show better specificity results than sensitivity. Additionally, the ellipses around each study, representing the confidence intervals, are closer to the central point and show a narrower elliptical area along the specificity axis. This elliptical area suggests higher and more precise specificity compared to sensitivity.

The studies that deviate most from the main group, particularly those in the upper right quadrant, focus on the anterior, posterior tibial, and fibular arteries. These studies show lower specificity and larger confidence intervals, indicating poorer performance in the diagnostic test.

Finally, when evaluating the SROC graph curve, we see that the confidence interval is remarkably close to the result line. This indicates a high consistency of results, which is favored by the large number of samples compared.

**Figure 5:**
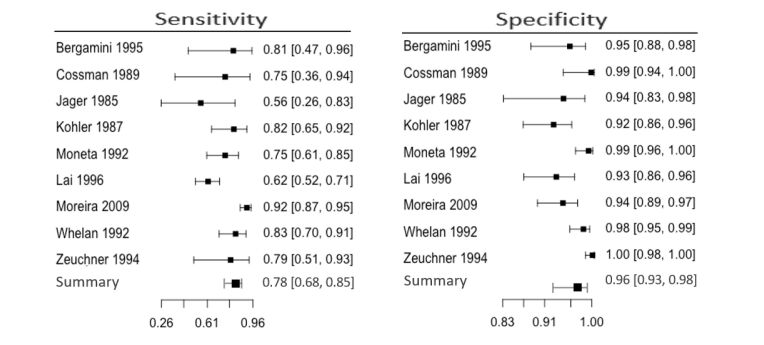
Meta-analysis results for the aorto-common femoral segment.

**Figure 6:**
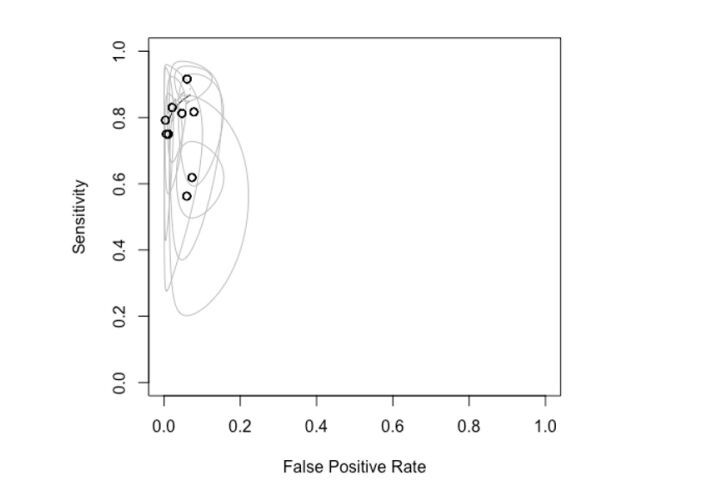
SROC for analysis of the aorto-common femoral segment.

Regarding the aorto-common femoral segment, based on a meta-analysis of 1924 arteries in nine studies, we conclude that sensitivity and specificity for diagnosis of arterial lesions were 0.78 (95% CI 0.68-0.85) and 0.96 (95% CI 0.93-0.98) respectively (Figure 5). Visual analysis of the forest plot and SROC graph reveals more significant heterogeneity and wider confidence intervals for sensitivity than specificity (Figure 6). The iliac was the most compared artery, mainly when the common and external segments were analyzed as unique arteries. The iliac had the best accuracy, while the common femoral artery had weaker performance.

**Figure 7:**
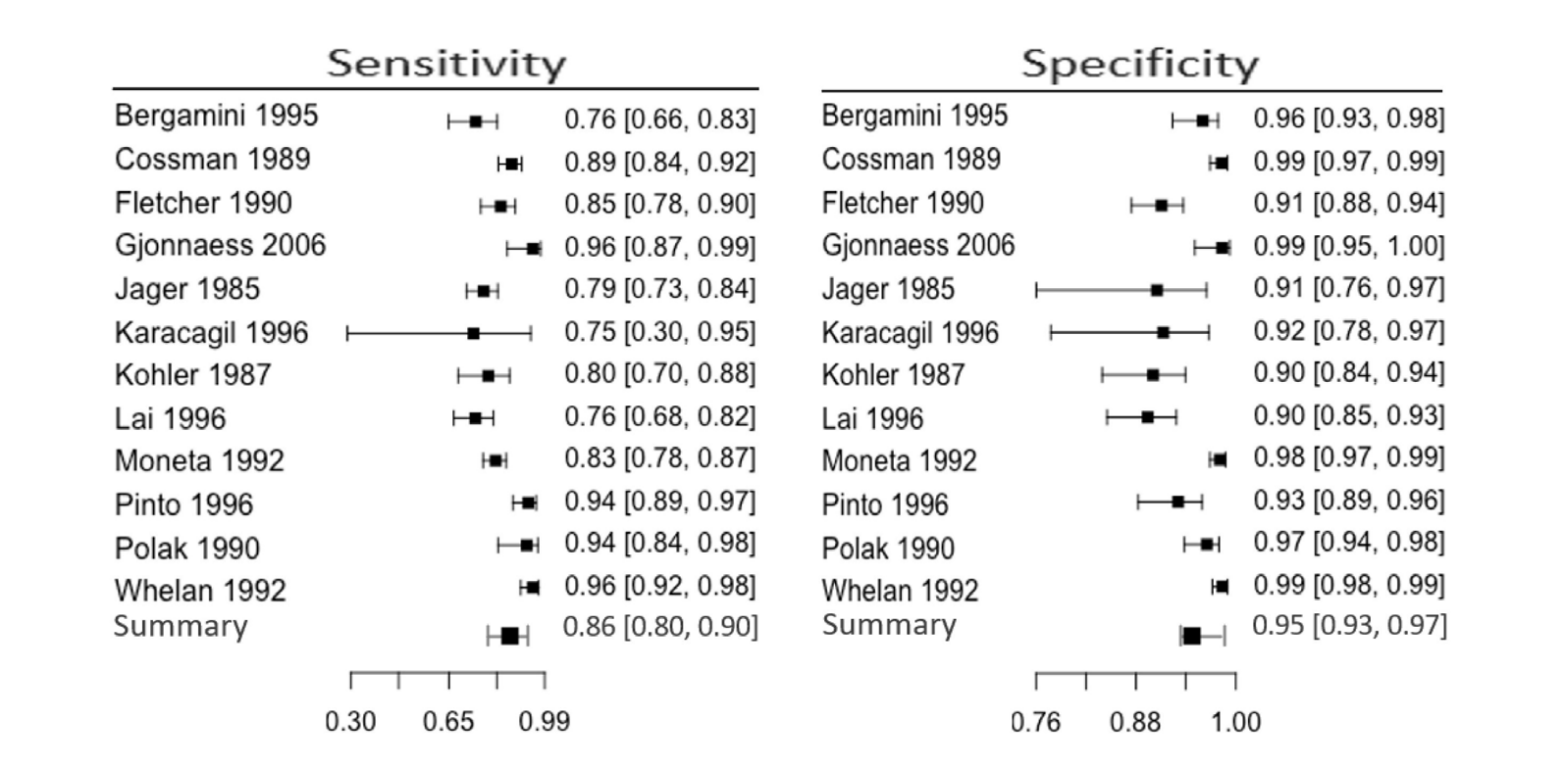
Meta-analysis results for the femoropopliteal segment.

**Figure 8:**
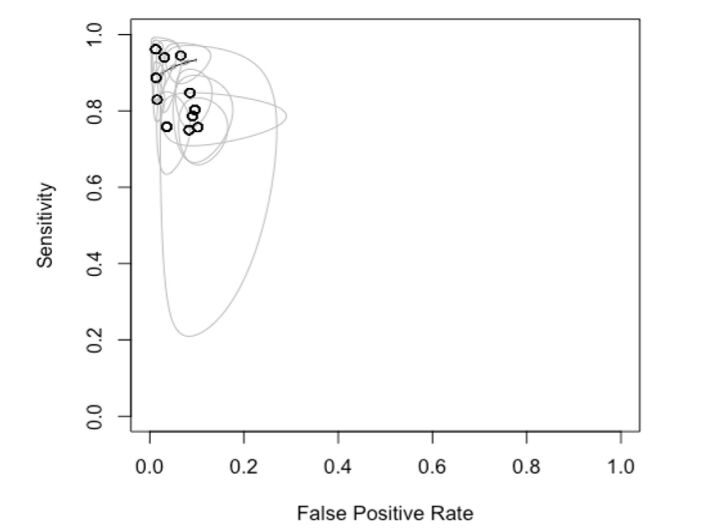
SROC for analysis of the femoropopliteal segment.

Regarding the femoropopliteal segment, based on a meta-analysis of 5000 arteries in twelve studies, we conclude that sensitivity and specificity for diagnosis of arterial lesions were 0.86 (95% CI 0.80-0.90) and 0.95 (95% CI 0.93-0.97) respectively (Figure 7). Visual analysis of the forest plot and SROC graph reveals more heterogeneity and wider confidence intervals for sensitivity than specificity (Figure 8). The femoral artery, and its segments, were the most compared in this review. The best accuracy was for the supra and infra-popliteal artery and the proximal third of the femoral artery, followed by the distal third of the femoral artery and the medial third of the femoral artery.

**Figure 9:**
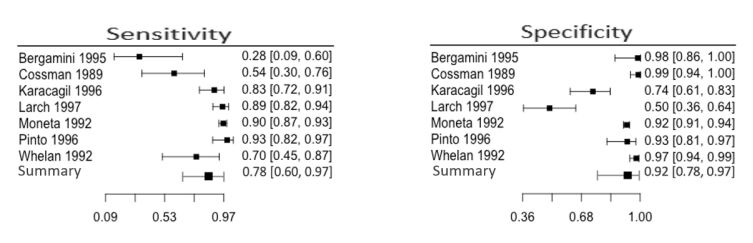
Meta-analysis results for the below-the-knee segment.

**Figure 10:**
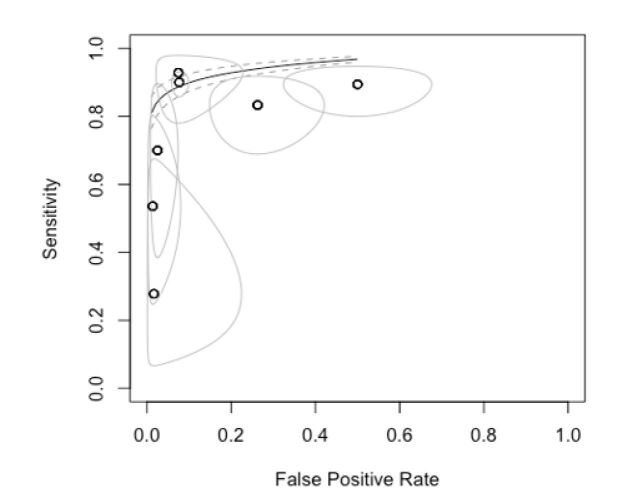
SROC for analysis of the below-the-knee segment.

For the below-the-knee segment, based on a meta-analysis of 2143 arteries in five studies, we conclude that sensitivity and specificity for diagnosis of arterial lesions were 0.78 (95% CI 0.60-0.97) and 0.92 (95% CI 0.78-0.97) respectively (Figure 9). Visual analysis of the forest plot and SROC graph reveals more heterogeneity and wider confidence intervals for sensitivity than specificity (Figure 10). The best results were for the anterior and posterior tibial arteries, followed by the tibiofibular trunk and fibular arteries.

## DISCUSSION

The femoropopliteal segment shows the best accuracy compared to the aorto-common femoral and below-the-knee segments. This is mainly due to its higher sensitivity, since specificity is similar for the aorto-common femoral and femoropopliteal segments. The highest accuracy was found in the supra and infra-popliteal arteries and the proximal third of the femoral artery.

With good performance in the femoropopliteal segment, DUS can reduce the need for invasive exams in claudicating patients. When DUS shows total occlusion of the femoral and popliteal segments, bypass surgery for the leg arteries is one of the first options for revascularization. However, this surgery is reserved for cases of critical limb ischemia, tissue injuries, and rest pain. Therefore, DUS is sufficient to delay invasive tests in claudicating patients, reserving them for more advanced cases.

The below-the-knee segment has the lowest accuracy, with the lowest sensitivity and specificity of all segments. Therefore, for patients with a regular popliteal pulse but decreased distal pulses, a negative DUS test may not be sufficient to rule out below-the-knee peripheral arterial disease.

We can use this information to assess the diagnostic accuracy of the method in the general population. For example, we know that the prevalence of PAD is 18% at age 50 and 29% at age 70. Transposing these values to a Fagan Nomogram, if the test is positive for the disease, we observe that the post-test probability is 80 and 87% for the respective ages above. Furthermore, if it is negative, the post-test probability of the disease is 2 and 5%, respectively. Therefore, these are robust numbers for diagnostic confirmation or exclusion using DUS, justifying its use for diagnostic complementation.

The review carried out by Collins et al.^5^ included 28 studies reporting results for DUS. We included only five in our review because of our pre-established diagnostic criteria. In our review, we used spectral flow velocity as a diagnostic instrument; this is a more objective criterion for diagnosing stenosis and occlusion, whereas B and Color Mode increase the heterogeneity of the results if used exclusively.^6^

Considering only stenosis > 50%, Collins et al.^5^ found that DUS had 71–100% sensitivity and 50–97% specificity for the above-the-knee segment. For the below-the-knee segment, sensitivity was 41–96% and specificity was 80–99%.

In our review, when grouping the arterial segments similarly, we found sensitivity and specificity of 82–91% and 94–98% for the above-the-knee segment. For the below-the-knee segment, sensitivity was 66–86% and specificity was 89–97%. Given the variability in Collins et al.^5^ sensitivity and specificity results, we can infer that our values were more accurate because we used more objective diagnostic criteria.

### Future study

Future studies should determine the accuracy of DUS exclusively for the diagnosis of stenosis and exclusively for the diagnosis of occlusion, given that there are specific, individual criteria for each. These studies should also evaluate patients with claudication separately from those with critical ischemia. The former show more minor impairment of the arterial circulation, thus decreasing the sensitivity for diagnosing lesions.

In addition, DUS is of greater importance for patients with claudication than critical ischemia. Those with critical ischemia need, a priori, to undergo more accurate tests like magnetic resonance imaging, tomography, or arteriography. It is necessary to demonstrate with quality and accuracy the connections between the distal arteries and the arteries of the foot to plan revascularization procedures.

Arteriography is currently the reference standard for diagnosing stenoses and occlusions, but it is essential to use the biplanar method. However, 40% of the studies in our systematic review employed single-plane arteriography, which may fail to diagnose lesions, thus erroneously increasing the number of false positives on the index test.

Future studies should explore the reasons behind the low sensitivity of DUS for detecting lesions in the arteries below the knee. This low sensitivity may be due to the small diameter of these arteries, bone interference with ultrasound waves, and the considerable length of arteries. The combined length of the anterior, posterior tibial, and fibular arteries increases the chance of missing lesions, which reduces the overall sensitivity of the exam.

Based on the review data and available technologies, future studies should explore two ways to improve the diagnostic performance of DUS.

First, use the ankle-brachial index (ABI) for the anterior and posterior tibial arteries during DUS. If no lesions are found in the femoropopliteal segment during the DUS test but the ABI is abnormal, this indicates a need for a closer examination of the tibial arteries.

Second, protocols should be established to adjust color Doppler settings to detect lower velocities. The anterior, posterior, and fibular arteries have lower velocities than the proximal segments, so fine-tuning the settings for lower velocity detection is essential. This adjustment helps identify aliasing, which can indicate stenosis.

### Limitations

Our study has some limitations. We were able to evaluate each of the segments proposed in the methodology. However, some results were compromised, mainly due to the low primary studies.

Most studies were classified as having a high and unclear risk of bias, so the meta-analysis results should be used cautiously in clinical practice.

## CONCLUSION

The accuracy of DUS, including spectral analysis, for detecting symptomatic PAD was 86% for sensitivity and 95% for specificity. The best results were observed for the femoropopliteal segment, while the below-the-knee segment had the weakest performance.

Based on our findings, DUS can be effectively used as a complementary test to confirm or exclude PAD in patients with clinical suspicion. The post-test probability values are particularly significant for diagnosing symptomatic PAD in elderly patients, who have a higher prevalence of the disease.

Furthermore, DUS results showing occlusion in the femoropopliteal segment may reduce the need for invasive contrast-based diagnostic tests. However, the performance of DUS is weaker for the below-the-knee segment, indicating the need for further studies to explore the reasons for this reduced accuracy. Future research could focus on improving DUS testing by incorporating the ABI and optimizing machine settings for detecting lower speeds.
